# Nanoscopy through a plasmonic nanolens

**DOI:** 10.1073/pnas.1914713117

**Published:** 2020-01-15

**Authors:** Matthew J. Horton, Oluwafemi S. Ojambati, Rohit Chikkaraddy, William M. Deacon, Nuttawut Kongsuwan, Angela Demetriadou, Ortwin Hess, Jeremy J. Baumberg

**Affiliations:** ^a^NanoPhotonics Centre, Cavendish Laboratory, Department of Physics, University of Cambridge, CB3 0HE Cambridge, United Kingdom;; ^b^Blackett Laboratory, Imperial College London, South Kensington Campus, SW7 2AZ London, United Kingdom;; ^c^School of Physics and Astronomy, University of Birmingham, B15 2TT Birmingham, United Kingdom;; ^d^School of Physics and CRANN Institute, Trinity College Dublin, Dublin 2, Ireland

**Keywords:** nanoscopy, plasmonics, super-resolution, single molecule, nanogap

## Abstract

Plasmonics now delivers sensors capable of detecting single molecules. The emission enhancements and nanometer-scale optical confinement achieved by these metallic nanostructures vastly increase spectroscopic sensitivity, enabling real-time tracking. However, the interaction of light with such nanostructures typically loses all information about the spatial location of molecules within a plasmonic hot spot. Here, we show that ultrathin plasmonic nanogaps support complete mode sets which strongly influence the far-field emission patterns of embedded emitters and allow the reconstruction of dipole positions with 1-nm precision. Emitters in different locations radiate spots, rings, and askew halo images, arising from interference of 2 radiating antenna modes differently coupling light out of the nanogap, highlighting the imaging potential of these plasmonic “crystal balls.” Emitters at the center are now found to live indefinitely, because they radiate so rapidly.

## Significance

Imaging of single to a few molecules has received much recent interest. While superresolution microscopies access subdiffraction resolution, they do not work for plasmonic hot spots due to the loss of positional information that results from plasmonic coupling. Here, we show how to reconstruct the spatial locations of molecules within a plasmonic hot spot with 1-nm precision. We use a plasmonic nanoball lens to demonstrate that plasmonic nanocavities can be used simultaneously as a nanoscopic and spectroscopic tool. This work opens up possibilities for studying the behavior of a few to single molecules in plasmonic nanoresonators, while simultaneously tracking their movements and spectral features. Our plasmonic nanolens is useful for nanosensing, nanochemistry, and biofunctional imaging.

Confining and coupling light to nanoscale objects is at the heart of nanophotonics (1). The possibility of imaging, localizing, and eventually manipulating nano-objects down to the level of single emitters is highly desirable for many applications and fundamental studies (2, 3). Approaches include the development of superresolution microscopies, such as stimulated emission depletion microscopy, structured illumination microscopy, stochastic optical reconstruction microscopy, and photoactivated localization microscopy (4, 5); however, there has also been intense interest in plasmonic nanostructures that utilize collective charge oscillations in noble metals to enhance the optical fields within a few nanometers (6). This extreme confinement of optical fields has significant implications for nanoscale sensing (7), advanced spectroscopies (8), biological applications (9), single-atom optics (10), and quantum (11, 12) and nonlinear photonics (13).

The tightly confined fields in plasmonic nanocavities enhance the fluorescence intensity of dye molecules, and their high optical density of states reduces the molecule’s emission lifetime (14–17). However, this enhanced emission comes at the cost of misrepresenting the position of individual molecules near the metallic structure (18–20). Emitters in the vicinity of a nanoparticle (NP) radiate into the far field via the plasmon mode and therefore appear displaced either toward or away from the NP center, by up to 100 nm (21). Similarly, the interaction of dye molecules with colloidal aggregates can produce surface-enhanced Raman scattering (SERS) signals spatially shifted from the NP photoluminescence (PL) (22). While superresolution microscopies access subdiffraction resolution, these techniques are currently impractical for plasmonic nanocavities, due to this loss of positional information associated with plasmonic out-coupling (23). Circumventing this limitation would allow plasmonic nanocavities to be simultaneously used as a nanoscopic and nanospectroscopic tool.

Here, we show how careful selection of the plasmonic architecture controls the confined optical modes, so that measurements of the far-field radiation patterns access near-field positional information. To generate high-quality, high-volume data, we explored the NP-on-mirror (NPoM) architecture, which consists of a Au NP coupled to its image charges on a Au mirror, from which it is separated by a self-assembled molecular layer (24, 25). This architecture forms extremely robust, reliable plasmonic nanocavities; is easily produced by using self-assembly, and allows for the study of thousands of identical structures on a single substrate. In this study, near-spherical Au NPs with diameters *D* = 60 or 80 nm are placed on flat Au mirrors after uniformly coating them with molecules of methylene blue (MB), each individually encapsulated inside a molecular container of cucurbit[7]uril (CB[7]). The CB[7] binds strongly to Au, with its molecular height ensuring a constant spacing of *d* = 0.9 nm between the Au NP and the Au mirror beneath (25–27), while also protecting the dye molecules and orienting them vertically.

## Results

### Theory and Simulations.

**Fig. 1.**
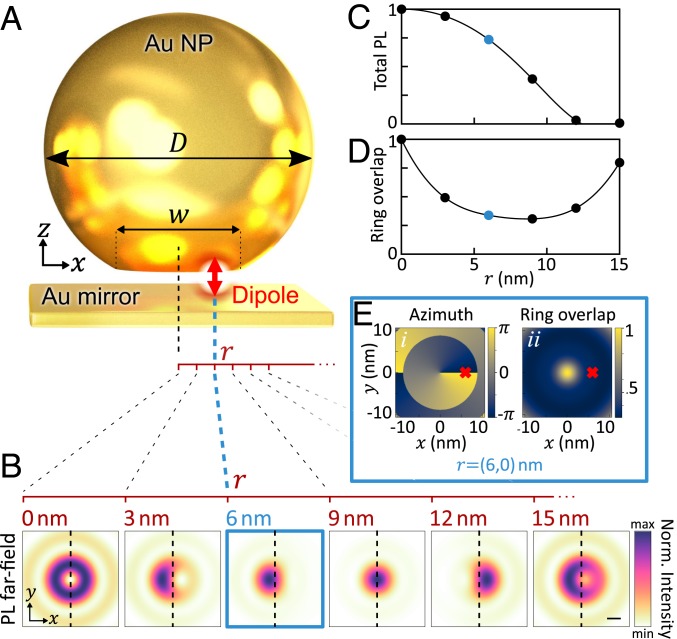
Simulated far-field images from a single emitter in the gap progressively shifted sideways. (*A*) Schematic plasmonic NPoM with vertically oriented dipole emitter placed at *r* up to 15 nm offset from center. (*B*) Simulated far-field real-space images (normalized, *λ* = 660 nm) after collection through high-NA objective (see text). norm., normalized. (Camera image scale bar: 100 μm.) (*C* and *D*) Emission intensity (*C*) and ring overlap (*D*) vs. radial location of emitter. (*E*) Extracted azimuthal weight (*i*; *ϕ*_*c*_) and ring-overlap integral (*ii*; *O*_*r*_), which reconstruct dipole position *r*. Red crosses give results for the emitter shifted to *x* = 6 nm.

Previous studies utilizing NPoMs [also known as particle-over-substrate, metal-insulator–metal waveguide, nanogap patch antenna (6) and equivalent to NP dimers, dumbbells (28), or homodimers (29)] suggest that light in the cavity is out-coupled through 1 of 2 antenna modes, either a transverse particle mode or a longer-wavelength, vertical-field gap mode (6, 30–32). Recent works show that emitters in the NPoM gap radiate dominantly through the gap mode, because of its stronger enhancement and radiative efficiency (33, 34). Because the optical field in the gap is mostly *z*-polarized (oriented as in Fig. 1), one would expect out-coupled light from these emitters to emerge at high angles from the dimer axis and thus produce ring-shaped distributions in the far field after collection through high-numerical-aperture (NA) microscope objectives.

Here, we show this is not the case and that emission from molecules within nanoscale plasmonic gaps depends on nonnegligible contributions from a large number of nanocavity modes. Furthermore, the coupling to each of these modes is highly dependent on the precise position of the molecules in the gap, which can therefore be inferred from the far-field spatial distribution of the out-coupled light. We first explored this complexity by solving Maxwell’s equations using finite element methods (FEMs; *Methods* and ref. 35). We obtained the far-field emission image of a dipole emitting at *λ* = 660 nm inside an 80-nm NPoM with gap *d* = 1 nm and facet diameter *w* = 20 nm, as it was shifted along the *x* direction by up to 15 nm (Fig. 1). Our calculations showed that the far-field ring emission seen for an on-axis dipole changed and became askew within a 1-nm lateral shift. Intriguingly, this nanobeaming tilted the far-field emission in the opposite direction to the emitter displacement under the facet (Fig. 1 *A* and *B*). The same emission patterns were confirmed with finite-difference time-domain simulations (*SI Appendix*, Fig. S1).

To invert these near-field transforms, the center of mass of each of the simulated images (*x*_*c*_, *y*_*c*_) was used to map the azimuthal orientation, *ϕ*_*c*_ = atan(*y*_*c*_/*x*_*c*_) + *ξ*_*c*_ (Fig. 1 *E*, *i*). The overlap integral *O*_*r*_ with the ideal ring distribution (Fig. 1*B*; *r* = 0) was used to quantitatively derive the fraction of ring-like emission in images at each emitter position (Fig. 1 *E*, *ii* and Fig. 1*D*). These then allowed a 1-to-1 mapping from (*ϕ*_*c*_, *O*_*r*_) extracted from measured images, to the position of a single dipole, seen as red crosses in Fig. 1*E* reconstructing *r* = (6, 0)-nm dipole position (for details on this method, including definitions of *ϕ*_*c*_ and *O*_*r*_, see *SI Appendix*, Fig. S2). We note that the total emission from the dipole (shown normalized in Fig. 1*B*) fell strongly when it was >9 nm from the central location (Fig. 1*C*), meaning that *r* > 10 nm was not observed.

**Fig. 2.**
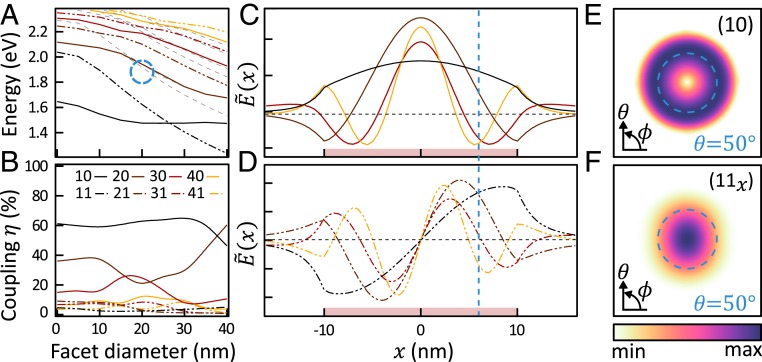
Plasmonic nanogap cavity modes of 80-nm NPoM with 1-nm gap and 20-nm facet diameter. (*A* and *B*) Mode energies (*A*) and out-coupling efficiencies (*B*) for the first 4 symmetric [solid lines (*lm*) = (10)–(40)] and asymmetric [dashed (11)–(41)] nanogap modes. Blue circle marks regime for facets of a typical *D* ∼ 80 nm NPoM. (*C* and *D*) Corresponding near-field mode amplitudes with a 20-nm facet (normalized; colors are as in *A*; dashed vertical line at *x* = 6 nm; extent of facet indicated by a pale red bar on the *x* axis). (*E* and *F*) Far-field angular emission patterns up to 50° collection aperture for (10) and (11_*x*_) emission.

To understand the peculiar behavior of these emission images, we characterized the quasinormal gap modes [found by using QNMEig (36)] and their out-coupling efficiencies using a near- to far-field conversion (37). The resulting angular emission when passed through appropriate Fourier filtering (*SI Appendix*, *Methods*) gave images matching those shown in Fig. 1*B*. As is well known for spherical NPoMs or dimers, the (10) gap mode dominate at low energies; however, as the gap facet widened (Fig. 2*A*), the energies of higher-order modes dropped and either crossed (20) or anticrossed (11) this mode (34) [for (*lm*) nomenclature, see *SI Appendix*, *Note*]. All other modes (gray) were dark, and, while the symmetric (*l*0) modes dominate emission (Fig. 2*B*), the (*l1*) modes emit through in-plane antenna dipoles (as seen in the angular emission pattern; Fig. 2*F*) which were 10-fold weaker but not negligible. We emphasize that since for such small gaps the gap fields are *z*-polarized, only the *z*-oriented dipole components contribute to emission.

For the 80-nm NPoMs used here with MB dyes (660 nm, 1.88 eV), facet sizes of 20 nm are typical (38), leading to operation in the regime marked by the dashed circle in Fig.2*A*. These emitted typically into a combination of {10, 20, 11} modes, depending not only on the spectral overlaps, but also on the spatial overlaps of the near-field modes with the emitter location (Fig. 2 *C* and *D*). For example, for an emitter at *x* = 6 nm (blue vertical dashed), the relative phase and amplitude of *x*-polarized (11_*x*_) and radially polarized (10, 20) antenna emissions combine to give the displaced spot (in the opposite direction as seen in Fig. 1*B*) shifted by several times the *λ*/NA resolution. The reversed spot displacement for *x* > 9 nm (Fig. 1*B*) can then be understood to arise from the change in sign of the (20, 21) modes near the facet edge (Fig. 2 *C* and *D*). These cross-overs did not vary substantially with the experimental range of facet sizes (*SI Appendix*, Fig. S3). Placing a plasmonic Au sphere on top of an emitter thus acts as a nanolens or plasmonic refracting globe, capable of expanding the resolvable field of view into the nanoregime.

### Experimental.

**Fig. 3.**
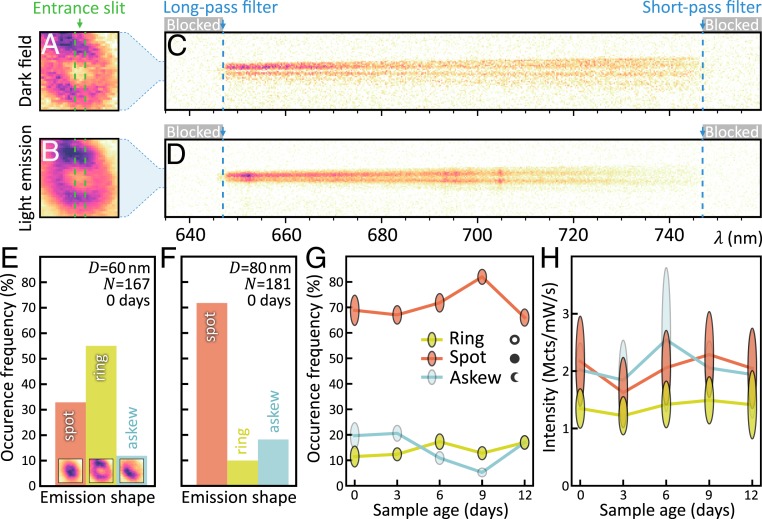
Real-space scattering and emission images from single NPoMs. (*A* and *B*) Spectrally integrated (647–747 nm) images of white-light dark-field scattering and light emission (in “ring” state). (*C* and *D*) Spectrally resolved emission through a vertical cross-section formed by the entrance slit (dashed lines in *A* and *B*) showing ring profile for both broadband PL and sharp SERS lines. (*E*–*G*) Relative occurrence of each shape (*E*, *Insets*) for *D* = 60-nm (*E*) and 80-nm (*F* and *G*) NPs. (*H*) Integrated emission intensity of 80-nm NPs, as sample ages after initial preparation. Vertical ellipses give SEs of fractions (*G*) and intensities (*H*) from *N*(*t*) NPs.

To observe this nanolens effect, we alternately recorded dark-field scattering and light emission from the same NPoM over time. To efficiently excite the gap mode, we used a radially polarized continuous-wave laser with a wavelength of 633 nm and power density of 150 μW⋅μm^−2^ at the focus. Samples created across large areas (4 × 4 mm^2^; *Methods*) showed consistent dark-field scattering spectra, with the lowest (10) mode centered at *λ*_10_ ∼ 730 nm (below and *SI Appendix*, Figs. S4 and S5), close to the MB dye emission at 690 nm. Hundreds of NPs were individually imaged and spectroscopically analyzed (24, 25), with the emitted light spatially magnified (∼3,500 times) onto the entrance slit of a monochromator after spectrally filtering out the 633-nm excitation laser (Fig. 3). The dark-field image from white-light scattering off each NPoM was always a ring (Fig. 3*A* and see Fig. 5 *A*–*D* and *SI Appendix*, Fig. S4), because *z*-polarized gap modes dominate the in-/out-coupling (Fig. 2*B*), converting the multimode excitation into a single spatial mode upon reradiation. Slight asymmetries in the dark-field images were mostly due to the polarization sensitivity of our optical system, leading to slightly greater intensity along the vertical axis relative to the horizontal axis. Inelastic light emission, on the other hand, was a combination of PL, surface-enhanced resonant Raman scattering (SERRS), and background electronic Raman scattering from the Au and gave very different spatial shapes. Indeed, sometimes rings were observed (Fig. 3*B*), but also partial rings (termed “askew,” presenting a partial halo surrounding an off-center dark central spot), as well as bright “spots” which had no dark center. For each category, the inelastic emission showed similar emission spectra (Fig. 3*D*), implying that they all originated from the same dye molecules. Samples with identical CB[7] spacers but without dyes showed negligible emission, but identical dark-field spectra (*SI Appendix*, Fig. S6), confirming that the dye emitters indeed formed local light sources.

Categorizing >3,000 identically prepared particles revealed that each category of far-field shape occurred with a consistent probability. While immediately after sample preparation (0 days), the larger, 80-nm NPoMs showed mostly spots with only 25% circular or askew rings (Fig. 3*F*), this observation was reversed for 60-nm NPoMs, which gave >65% rings (Fig. 3*E*). This extreme sensitivity to NP size suggests the strong influence of interacting gap modes, discussed further below. While nanoscale faceting [which changes with aging (39)] could influence emissive nanolensing, we observed little change in these statistics for >10 d (Fig. 3 *G* and *H*), including little decrease in emission intensity (each time probing slightly different regions of the sample). This suggests that, in the dark, both plasmonic and dye components of the construct are stable. These results should be compared to antenna-directed angular emission from dyes in the vicinity of other nanostructures. For example, high directivity of emission from Atto740-dyes near Au nanorods has been observed (40), with emission patterns showing sensitivity to dye position on the order of 100 nm. Emission from Cy5.5 dye molecules near Au nanoislands shows similar sensitivity (21), as does the emission of quantum dots coupled to nanoscale Yagi–Uda structures (41).

**Fig. 4.**
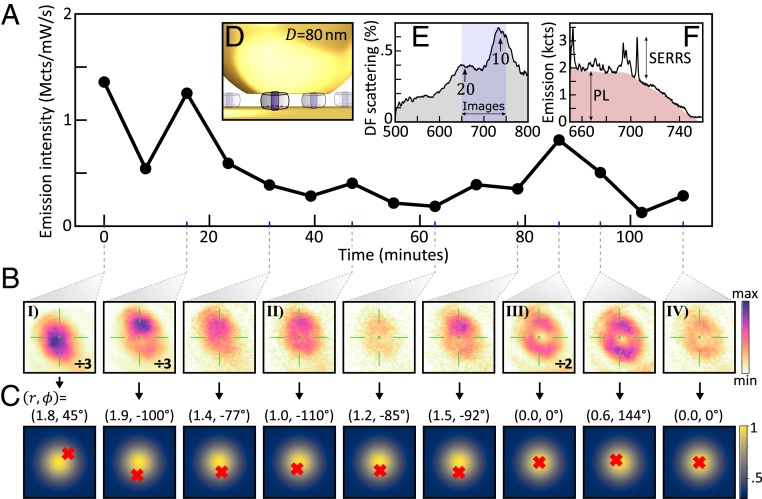
Time evolution of real-space emission from a single 80-nm NPoM. (*A* and *B*) Integrated intensity (*A*) and corresponding real-space spectrally filtered emission images (*B*) at times marked; green reticle is at dark-field ring center. (*C*) Ring-overlap integral map from COMSOL simulations (for |*x*|, |*y*| < 5 nm), with reconstructed coordinates (*r*[nm], *ϕ*[°]) of weighted emitter position in the NPoM cavity marked with a red cross (see text). Note gradual movement toward center of the facet over time (see text for discussion). (*D*) Schematic MB dyes (blue) in CB within plasmonic gap. (*E*) Dark-field elastic scattering spectrum, with emission detection range shaded. (*F*) Emission spectrum showing integrated emission is dominated by dye PL.

Prolonged observation on a single NPoM, however, revealed that the far-field emission intensity and distribution of a particle was not fixed, but instead varied with time under illumination (Fig. 4 *A* and *B*). For example, an NP which initially emits into a spot can gradually change to produce a ring-shaped emission (Fig. 4*B*), or vice versa (*SI Appendix*, Figs. S8–S10). Many particles were observed to wander between all 3 different scattering distribution types, at times returning to a previously observed type more than once (Fig. 4 *A* and *B* and *SI Appendix*, Fig. S8). This evolution was accompanied by a gradual reduction in the overall emission intensity, although the intensity tended to a steady value that was ∼30 times higher than the background from a cavity without dyes, even after 2-h irradiation (*SI Appendix*, Figs. S6 and S7). This implies that full bleaching was never observed, although full recovery of emission to initial levels was also never observed. This was confirmed in a second experiment by irradiating a single particle for 4 cycles, each comprising 15-min illumination and 5-min rest in the dark, with little to no recovery in emission observed following each rest period.

These changes in the emission intensity and far-field profile of individual plasmonic nanocavities suggest that emitters at different positions within the gap out-couple through different cavity modes at different times (Fig. 4*E*; *Discussion*). Since CB[7] forms monolayers with 0.24 molecules⋅nm^−2^ (42), a 1:1 molar ratio of CB[7] to MB gives ∼75 emitters within these gaps (for 80-nm NPoMs with 20-nm facets). Given the assembly protocol, these are expected to be randomly distributed across the nanogap (Fig. 4*D*), although only ∼19 contribute strongly to the (10) mode emission. We note that while complex changes to the SERRS spectra sometimes appear over time (*SI Appendix*, Fig. S11) with changing Raman peak positions and intensities, we stress that the integrated emission was dominated (>85%) by the PL (Fig. 4*F*).

**Fig. 5.**
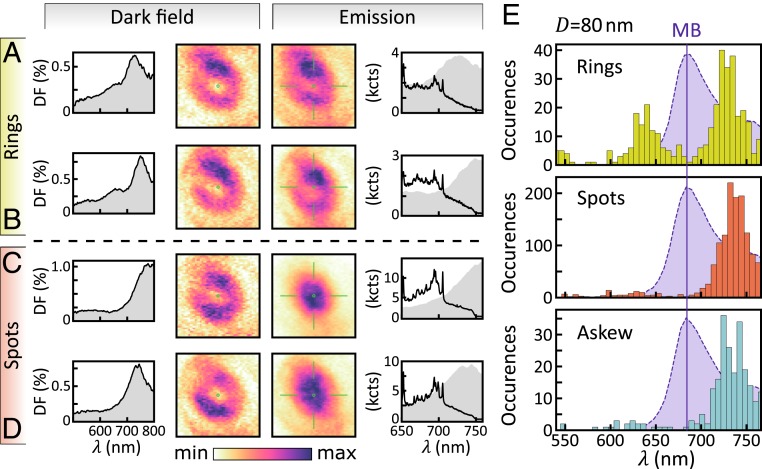
Comparison of ring and spot emission from 80-nm NPoMs. (*A*–*D*) From left to right: Dark-field spectra, dark-field image, emission image, and emission spectra for NPoMs exhibiting rings (*A* and *B*) and spots (*C* and *D*). (*E*) Analysis of scattering spectra peak central wavelengths classed by shape, for 1,602 NPoMs. Shaded gray curves in emission spectra (*A*–*D*) are same dark-field (DF) spectra. Purple shaded curves in *E* show the MB dye emission in solution.

Since the NP diameter sets the facet width which controls the spectral tuning of the NPoM gap modes (38), the prevalence of rings (for *D* = 60 nm) vs. spots (for *D* = 80 nm) suggests the crucial importance of precisely which range of gap modes the molecules emit into (Fig. 3 *E* and *F*). Exploring more carefully the differences between 80-nm NPoMs which originally show rings vs. spots (Fig. 5 and *SI Appendix*, Fig. S4) provided further evidence. While NPoMs with spot-shaped emission generally gave dark-field scattering spectra dominated by the (10) resonance (Fig. 5 *C* and *D*), the NPoMs with ring-shaped emission showed also the higher-energy (20) resonance around 640 nm (Fig. 5 *A* and *B*). This was confirmed by extracting the resonance peak positions from the scattering spectra of 1,602 NPoMs, showing that this trend was robust (Fig. 5*E*). Similar scattering spectral changes accompanied the evolution of shape during time-resolved experiments (Fig. 4 and *SI Appendix*, Figs. S8–S10). The (20) mode increases out-coupling at shorter wavelengths, thus broadening the spectral emission from ring-NPoMs compared to spot-NPoMs. Askew NPoMs had similar emission spectra to the spot-NPoMs (*SI Appendix*, Figs. S8*C* and S10*C*).

## Discussion

Emission into a ring implies that dyes near the central axis of the NP must dominate (Fig. 1 and *SI Appendix*, Figs. S3 and S12). Experiments showed that these rings emerge with most 60-nm NPoMs (*λ*_10_ ∼ 680 nm) and for those 80-nm NPoMs (*λ*_10_ ∼ 730 nm) with a larger facet [*w* > 25 nm, estimated from the (20) resonance position]. With smaller facets and smaller NPs, the (10) mode is more tightly confined (nearly 3-fold closer to the axis when *w* reduces from 20 to 0 nm), so that only dyes within 2 nm of the facet center experience strong Purcell factor enhancements and high quantum yield of emission (43) (Fig. 1). This explains the 60-nm NPoM data, since the smaller size and facets of these NPs imply emission from ∼3 centrally positioned MB dyes, where the MB emission is tuned resonantly to the lowest gap plasmon. For comparison, only dyes within 3 nm of the facet center produced rings in 80-nm NPoMs, which at a surface packing density of 0.24 molecules⋅nm^−2^ (42) implies emission from <7 molecules. After bleaching (such as observed in Fig. 4), this reduces to ≤3 molecules.

The bright spots seen initially from the 80-nm NPoMs (as in Fig. 5 *C* and *D*) must arise from multiple dyes at different positions ranging across the facet. In this situation, the coordinates given in Fig. 4*C* must be interpreted as the average position of emitters, indicating the location of the greatest dye concentration. For a *w* = 20-nm facet, the most likely dye location for out-coupling the PL is at *r* = 4.5 nm for the (10) mode, which will give a spot in the far field (*SI Appendix*, Fig. S13). This increases to *r* = 9.1 nm for the (11) mode, which is spectrally resonant with the PL, although it out-couples 25 times more weakly (Fig. 2*B*). Either coherently or incoherently summing the emission from multiple dyes randomly placed under the NPoM indeed predicts spot-like emission (*SI Appendix*, Fig. S12).

The full interplay of facet size and emitter position is complex in producing different emission shapes. However, askew ring-like distributions always indicate an off-center emitter, with the degree of asymmetry dependent on the emitter’s radial coordinate and thus allowing the nanoscale position of the dye to be estimated. When the facet size is known, along with the maximum emission intensity per dye (when placed at the nanocavity center), a full inversion of the patterns can yield the relative position of each dye. This is possible due to the fundamental symmetries of the (10), (20), and (11) modes in the near and far fields, which is a feature of not only faceted spherical NPoMs, but also, for example, nanocubes-on-mirror (34). A small change in gap size does not change our inversion technique and only slightly updates the appropriate mapping (*SI Appendix*, Note and Figs. S17 and S18). Similarly, different NP shapes lead to modified mappings from those shown in Fig. 1*E* and *SI Appendix*, Fig. S2.

However, because the emitters can be strongly coupled by the plasmonic gap modes (25, 44, 45), emitters can be coherently coupled together and jointly emit into the plasmonic gap modes which subsequently radiate, further complicating the picture outlined here. To avoid this, the samples used here are in the weak coupling regime, since there is a large detuning between the dye emission peak and the cavity resonance of the 60- and 80-nm NPoMs compared to the 40-nm NPoMs used in ref. 25. The smaller, 40-nm NPs have a smaller scattering strength (∝ *D*^6^) and have fewer molecules in the nanogap due to their smaller facet size (*w* ∼ 6 nm), making the emission too weak to resolve spatially. Measurements on strongly coupled systems would, however, be extremely interesting for the observation of spatial coherent interactions between the emitters and nanocavity.

The MB dye was chosen for our experiments not only because it is tuned into the weak coupling regime, but because it is the longest-wavelength dye that fits within a CB[7]. Any further detuning of the dye emission peak from the cavity resonance makes emission too weak to resolve spatially. However, since simulations show that a different emission wavelength simply leads to the spot/ring transitions occurring at different emitter positions to those observed with a 660-nm wavelength, tuning the emission wavelength is completely analogous to changing the NPoM size, which is more easily studied experimentally.

The progression from spots to rings seen in time (Fig. 4 and *SI Appendix*, Figs. S8–S10) can be understood from the progressive bleaching of different dyes and rules out the possibility of different emission patterns being due to nanometer-scale surface-roughness features on the Au mirror (32). However, it also points to a surprising feature, since the convergence to rings at longer times implies that the dyes which last longest are always at the center of the facets. This suggests that either the narrow gaps physically protect molecules on the inside from photochemical attack and/or that the Purcell factors for the dyes at the center (*F*_*P*_ > 3,000; ref. 43) are so high that the molecules emit their photons before any chemical attack or intersystem crossing is possible. While encapsulation of dyes in CB[7] is known to partially protect them from bleaching (46, 47), this is unable to explain the extreme stability observed here.

One possible explanation for the sporadic transient revivals of emission could be lateral diffusion of dyes in and out of the hot spots. However, this is not the case, since fewer than half the number of rings were observed when introducing dye solution only after the NPoM constructs were assembled, suggesting limited migration of MB molecules toward the center of the NPoM cavity. On-site reorientation of the MB dyes from in-plane (dark) to *z*-oriented (bright) could cause such revivals, but would be prevented by the well-defined CB[7] binding orientation (Fig. 4*D*). Another hypothesis for the revivals is migrating Au adatoms that can trap light into picocavities close to single molecules (10, 48) or transient defects in the Au facets that depress the local plasma frequency, resulting in large enhancements of electronic Raman emission (49). Such transient phenomena can also lead to the SERRS peaks observed (*SI Appendix*, Fig. S11), but will demand further enhancements of our spatiotemporal nanoscopy technique, which is shot-noise limited.

One intriguing test of our emitter position reconstruction method is to locate positions of molecules placed at known positions in the gap, for example, using DNA origami (12, 43). However, our attempts to do this have shown that the DNA layer within the NPoM introduces a fluctuating background intensity and increases the gap thickness to ∼5 nm, hence decreasing the dye signal intensity below the limit required for our spatial reconstruction imaging.

The construction of localized nanolenses formed of plasmonic nanogaps supporting many highly localized transverse modes does, however, offer a route to peer inside solvated molecule–metal interfaces under ambient conditions and resolves here how bleaching of molecules can be localized within a few nanometers. While in typical plasmonic constructs, only a single mode controls emission, in all plasmonic narrow-gap systems (such as dimers, patch antennas, and these NPoMs), the multiple gap modes can clearly yield spatial information when the gap modes are well understood.

An intriguing scenario would be to use Au nanoconstructs as nanolenses to reconstruct deep subwavelength images in real time to track the movement of emitters inside this nanogap. Because these nanogap quasinormal modes form a complete basis set, improved localization requires only broader spectral emission into many modes, together with interferometry (imaging) in the far field. However, the symmetry of the currently faceted NPoMs or NP dimers leads to many modes being dark (*m* > 2 states), which can limit azimuthal information [though broken by noncylindrical symmetry of the NP (50)], while emission into these 3 antenna modes (the *z*-dipole in Fig. 2 *C* and *E* and *x*,*y*-dipoles in Fig. 2 *D* and *F*) limits direct Fourier imaging approaches without the basis-state reconstruction discussed above. Approaches such as localization microscopy (4) using frame-by-frame images of each photon emitted can be combined with the techniques here (as in Fig. 4). Already, these nanocavity gap modes can deliver nanometer precision from single frames for the location of single molecules and resolve how multiple active emitters are distributed and change spatially over time.

## Methods

### Sample Preparation.

Sample preparation began with fabrication of the Au mirror substrate by a template-stripping process. A silicon wafer was coated with a thin layer of Au (100 nm) by thermal vapor deposition. A second silicon wafer was then scored with a diamond scribe and broken into many small pieces, which were then fixed to the Au surface on the first wafer by using epoxy (Epo-Tek 377). After curing (by heating to 150 °C and then slowly cooling to room temperature), these pieces can be stripped off the first wafer as needed, by gentle application of shear force using a pair of tweezers. The Au surface on silicon freshly stripped in this manner is very clean and exceptionally flat [rms roughness of 0.23 nm (38)]. Silicon pieces still affixed to the Au layer on the first wafer can be stored at room temperature in air indefinitely and stripped off as needed.

Fabrication of the NPoM structure with CB[7]:MB host–guest complex was then carried out by preparing a 1 mM solution of MB and a 1 mM solution of CB[7] and mixing these together, allowing encapsulation of the MB guest molecules inside the CB[7] cavities. A freshly stripped piece of template-stripped Au was then allowed to soak in this solution overnight. Finally, a small droplet of citrate-stabilized Au NP solution (BBI Solutions) was then pipetted onto the prepared piece of template-stripped Au and allowed to rest for a few seconds. Excess NPs were flushed from the Au with deionized water, and the surface was blown dry with a nitrogen gun. Samples prepared in this manner can be stored under nitrogen flow for at least 2 weeks, with little observed change in spectra or numbers of each emission shape (Fig. 3*G* and *SI Appendix*, Figs. S14 and S15).

### Collection of Spectra and Far-Field Scattering Profiles.

Samples were studied by using the experimental setup shown in *SI Appendix*, Fig. S16 with a detailed description in *SI Appendix*, *Experimental Setup*. Samples were illuminated with white light from a halogen lamp and were imaged in dark field through a 100× dark-field/bright-field objective using a charge-coupled device (CCD) camera. These dark-field images were used in conjunction with a motorized stage to automatically center the view on a target NP for analysis. Computer vision and automation for this purpose were enabled by Python and the Open Source Computer Vision Library. After centering, a dark-field spectrum was taken under the same white-light illumination by using a fiber-coupled spectrometer (*SI Appendix*, Fig. S5). A second, magnified, dark-field image of the target NP was taken on a second electron-multiplying CCD camera by using the zero-order reflection of a grating monochromator after passing through a series of prefilters (Thorlabs catalog nos. FEL0650 and FES0750; *SI Appendix*, Fig. S16) to limit collected light within the 650- to 750-nm wavelength range. The white light was then shuttered, and a radially polarized HeNe laser was turned on, allowing excitation of the MB molecules in the sample at the pump wavelength of 633 nm. The emission was collected and imaged in the same configuration as the dark field, by using the zero order of the monochromator. Finally, the monochromator grating was rotated to first order, and the spectrum of the emission was collected. The computer then automatically moved the motorized stage to the next particle, and the process was repeated (hundreds of NPoMs in each single experiment). We note that the weak light emission from individual NPoMs of 1 k-counts/mW/s integrated or 0.5 counts/mW/s/pixel in images means that integration times of 10 s are required to adequately discriminate the different shapes, hence integrating over any more rapidly fluctuating phenomena such as the recently described picocavities (10, 48, 51).

### COMSOL Multiphysics Simulations.

The optical properties of the NPoM system were simulated by using the FEM to solve Maxwell’s equations (COMSOL Multiphysics, Version 5.4). The Au NP was modeled as an 80-nm-diameter sphere with a flat lower facet 20 nm in diameter. The permittivity of Au was modeled by a 2-pole Lorentz–Drude permittivity $\varepsilon \left(r;\omega \right)=\varepsilon_{0}\varepsilon_{\infty }\left(1-\sum_{i=1}^{2}\omega_{p,i}^{2}/\left(\omega^{2}-\omega_{0,i}^{2}+i\omega \gamma_{i}\right)\right)$, where *ε*_∞_ =  6, *ω*_*p*,1_ = 5.37 × 10^15^ rad/s, *ω*_0,1_ = 0 rad/s, *γ*_1_ = 6.216 × 10^13^ rad/s, *ω*_*p*,2_ = 2.2636 × 10^15^ rad/s, *ω*_0,2_ = 4.572 × 10^15^ rad/s, and *γ*_2_ = 1.332 × 10^15^ rad/s. The gap spacer was modeled as a 1-nm-thick dielectric layer with refractive index 1.45, while the background material had a refractive index of 1.

In Fig. 1, a point electric dipole emitter was placed in a NPoM gap at different radial coordinates, and the simulations were carried at a wavelength of 660 nm. The plasmonic nanogap cavity modes (Fig. 2) were modeled as quasinormal modes (QNMs) described with complex eigenfrequencies. The QNMs were calculated by using QNMEig (36), an open-source program based on COMSOL. The far-field emission patterns of the emitter and of each QNM were obtained by using RETOP (52), an open-source code for near-to-far-field transformations of generalized guided waves. Finally, the out-coupling efficiency of each QNM was computed as the ratio of the far-field radiation power to the total dissipated power.

### Data Availability Statement.

All relevant data present in this publication can be accessed at https://www.repository.cam.ac.uk/handle/1810/299012. The source data underlying Figs. 1–5 are provided.

## Supplementary Material

Supplementary File
