# Supplementary material for: Nanoscopy through a plasmonic nanolens
Source: Proc Natl Acad Sci U S A. 2020 Jan 15;117(5):2275–81. doi: 10.1073/pnas.1914713117 (PMC7006646; doi:10.1073/pnas.1914713117)
Supplement: Supplementary File [file pnas.1914713117.sapp.pdf]

PNAS
www.pnas.org

Supplementary Information for

Nanoscopy through a plasmonic nano-lens

Matthew J Horton[1], Oluwafemi S Ojambati[1], Rohit Chikkaraddy[1], William M Deacon[1], Nuttawut Kongsuwan[2], Angela Demetriadou[2,3], Ortwin Hess[2,4], Jeremy J Baumberg[1]

[1] NanoPhotonics Centre, Cavendish Laboratory, Department of Physics, JJ Thompson Avenue, University of Cambridge, Cambridge, CB3 0HE, UK
[2] Blackett Laboratory, Imperial College London, South Kensington Campus, London, SW7 2AZ, UK
[3] School of Physics and Astronomy, University of Birmingham, Birmingham, B15 2TT, UK
[4] School of Physics and CRANN Institute, Trinity College Dublin, Dublin 2, Ireland.

Prof. Jeremy J. Baumberg, FRS
Email: jjb12@cam.ac.uk

This PDF file includes:

Supplementary text
Figures S1 to S18
SI References

Supplementary Information

Nanoscopy through a plasmonic nano-lens

Matthew J Horton[1], Oluwafemi S Ojambati[1], Rohit Chikkaraddy[1], William M Deacon[1], Nuttawut Kongsuwan[2], Angela Demetriadou[2,3], Ortwin Hess[2,4], Jeremy J Baumberg[1]

[1] NanoPhotonics Centre, Cavendish Laboratory, Department of Physics, JJ Thompson Avenue, University of Cambridge, Cambridge, CB3 0HE, UK

[2] Blackett Laboratory, Imperial College London, South Kensington Campus, London, SW7 2AZ, UK

[3] School of Physics and Astronomy, University of Birmingham, Birmingham, B15 2TT, UK

[4] School of Physics and CRANN Institute, Trinity College Dublin, Dublin 2, Ireland.

Methods: Simulations of a dipole emitter in a NPoM

A) FEM

In the FEM simulations performed using COMSOL, the optical fields are recorded on a two-dimensional plane in the space domain at a distance Δz=150 nm above the mirror surface. These fields are then propagated into the far-field and projected into real-space using a plane-wave approximation, accounting for the effects of the collection optics. This is done by first taking the discrete Fourier transform of the complex recorded field $E(x, y)$, in order to obtain an image of the field in the spatial frequency domain. This result is then multiplied by $\exp\{i(-\Delta z)k_z(x, y)\}$, where $k_z(x, y) = \sqrt{(2\pi/\lambda)^2 - k_x^2 - k_y^2}$ in order to obtain the spatial frequency domain image of the field in the focal plane of the objective. In order to account for the numerical aperture of the objective, this result is multiplied by a k-plane mask $\frac{1}{2}\left(1 - \tanh\left(\left(\sqrt{k_x^2 + k_y^2} - k_c\right)/k_s\right)\right)$, where $k_c = (2\pi/\lambda)\sin(50°)$ and $k_s = 0.1k_c$, before taking the inverse Fourier transform to recover the fields at the detection plane (in the spatial domain). The squared norm of these fields then yields the images shown in Fig.1b and supplementary Fig.S3.

1B) FDTD

To confirm the COMSOL results, the CB:MB in NPoM system is also simulated using a finite-difference time-domain Maxwell solver (Lumerical FDTD). The simulation is set up assuming an 80 nm spherical nanoparticle with a 20 nm diameter circular bottom facet. Material data used for the gold is taken from Johnson and Christy, and the CB:MB layer is assumed to have a refractive index of 1.4 and a thickness of 0.9 nm.

Simulations of an 80 nm spherical NPoM with a flat bottom facet of a range of sizes between 0 nm and 34 nm are designed to replicate as closely as possible the system used in the experiments. Dimensions of the particle, particle facet, and gap size are set correspondingly, and complex wavelength-dependent refractive indices are selected to match each material used. To simulate a molecule emitting in the NPoM gap, we use a broad-spectrum vertically-oriented dipole source placed in the plasmonic hot-spot. The

same filtering applied to the results of the COMSOL simulations is then applied here in order to obtain the images shown in supplementary Fig.S1.

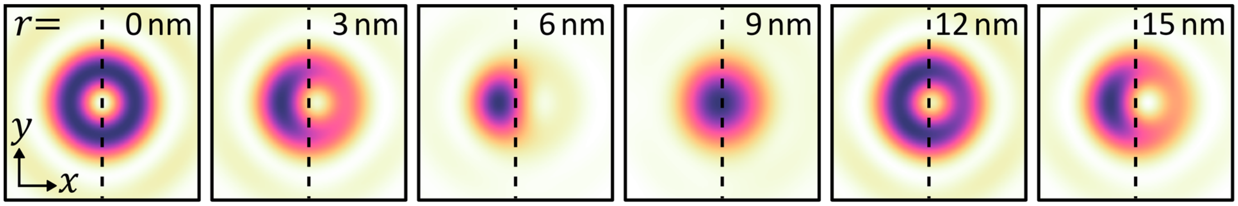

Fig.S1 | Far-field real-space images (normalized) simulated using FDTD after collection through an 0.9NA objective. The images confirm those obtained using the FEM method (Fig.1b).

Recovering emitter position from dark field and photoluminescence images

The method used to localize a single emitter within the cavity of each NPoM is derived from the results of modelling the NPoM structure numerically. This is done using either the FDTD model produced in Lumerical, or using the FEM model produced in COMSOL. The emitter positions obtained from either model agree to within <1 nm for emitter positions within a radial coordinate r<10 nm of the cavity centre. For emitter positions beyond 9 nm of the cavity centre, the emission intensity drops off rapidly for most NPoM geometries (supplementary Fig.S3). The spatial distribution of the emission here begins to resemble that observed at smaller values of r (supplementary Fig.S3 r=15 nm vs 3 nm) and hence it becomes less possible to unambiguously determine the position of the emitter. However since such highly displaced emitters give very weak light intensities, this has little effect on our results.

The procedure for extracting the position of a single emitter under each NPoM is outlined in supplementary Fig.S2.

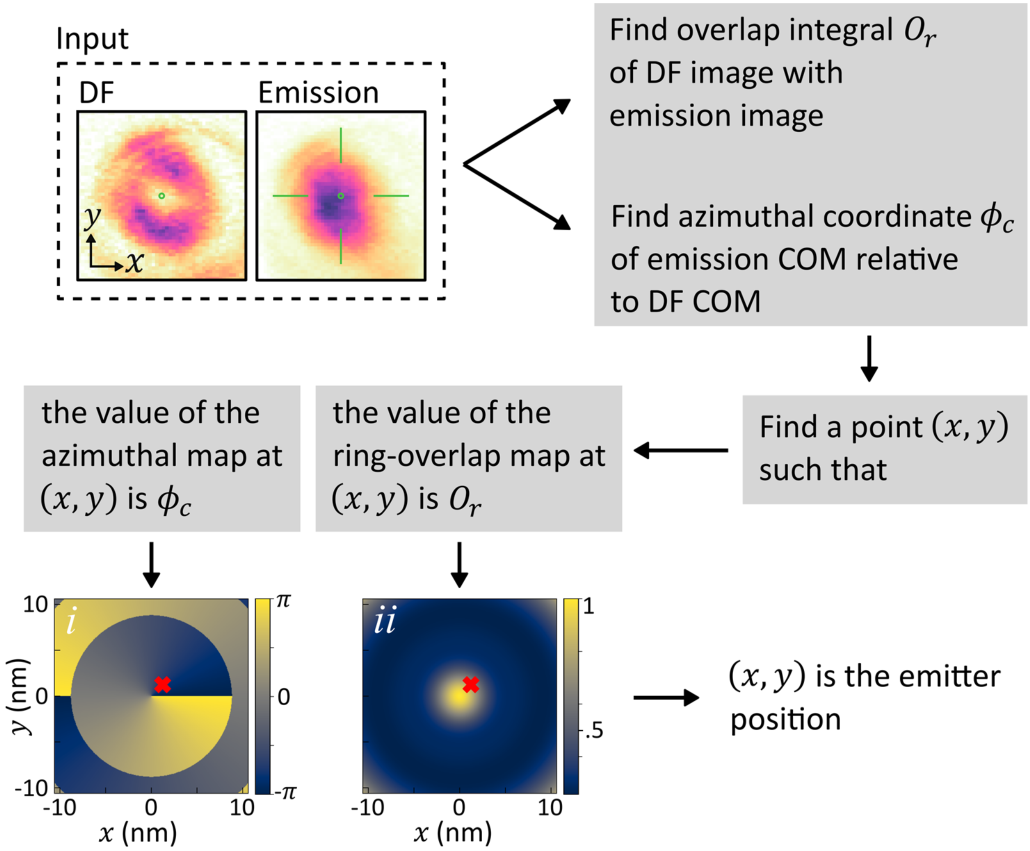

Fig.S2 | Methodology for recovering the position of an emitter in the NPoM gap using image of dark field scattering and image of the light emission (PL). The overlap integrals of a ring shaped distribution with the distributions obtained from emitters placed at different distances from the centre of the NPoM gap are computed using COMSOL simulations. These are used to generate a map where the radial coordinate of the emitter can be looked up given the same overlap integral calculated using experimental data. The azimuthal coordinate is simply obtained from the azimuth of the offset of the centre of mass of the PL image relative to the centre of mass of the dark field image.

First, the centre of mass of both the DF and the emission images is computed. The azimuthal coordinate of the emission centre of mass relative to the dark field centre of mass is then compared against an interpolated lookup table of the azimuthal coordinates for different emitter positions, generated from the simulation results. This yields the azimuthal coordinate of the emitter. Then, utilizing the fact that the DF spatial distribution of a NPoM is always ring-like, the overlap integral of the DF and emission is computed, and used as a measure of the degree to which the NPoM emission is ring-like (vs. spot-like). As before, this value is compared against an interpolated lookup table generated from the simulation results. This yields the radial coordinate of the emitter, completing the localization process.

The azimuthal lookup table $\phi_c(x, y)$ is generated from the simulations by taking the x coordinate of the centre of mass of the calculated emission image ($x_c(x)$) for a few dipole positions (at x=0, 3, 6, 9, 12, and 15 nm as shown in Fig.1b) along the positive x-axis (corresponding to a dipole azimuthal coordinate ϕ_d=0°). $x_c(x)$ is then linearly interpolated and passed through a threshold filter yielding 1 for x_c≤0 and 0 for x_c>0, thus indicating the domains where the emission beaming is in the direction respectively opposite or identical to the direction of displacement of the dipole. The values of the lookup table on the positive x-axis are then assigned to $\xi_c(x_c)$=180° wherever x_c≤0 and $\xi_c(x_c)$=0° wherever x_c>0. Finally, the rest of

the lookup table is filled exploiting the cylindrical symmetry of the NPoM geometry, by simply adding the azimuthal coordinate of the dipole $\phi_d(x,y) = \tan^{-1}(y/x)$ to the values of $\xi_c(x_c)$, so that $\phi_c(x,y) = \tan^{-1}(y/x) + \xi_c\left(\sqrt{x^2+y^2}\right)$.

The ring-overlap map $O_r(x,y)$ is similarly generated from the simulations by first computing O_r^{x+} at each of the dipole positions simulated (x=0, 3, 6, 9, 12, and 15 nm) on the positive x-axis. The method consists of taking the ring shaped emission image I_{ring} at x=0 (leftmost image in Fig.1b) and a second image $I_{emission}$ from among those in Fig.1b and computing:

$$O_r = \frac{1}{N}\sum_{u,v} \frac{I_{emission}(u,v)}{\max\limits_{u,v} I_{emission}(u,v)} I_{ring}(u,v)$$

where

$$N = \frac{1}{\max\limits_{u,v} I_{ring}(u,v)} \sum_{u,v} I_{ring}(u,v)^2$$

and u, v are the image coordinates in pixels. This is done taking $I_{emission}$ to be each of the images in Fig.1b in turn. The remaining values of O_r^{x+} are found by interpolation using a cubic spline. The full lookup table $O_r(x,y)$ is then filled by again exploiting the cylindrical symmetry of the NPoM geometry, and simply rotating O_r^{x+} about $(x,y) = (0,0)$.

COMSOL simulation of emission profile for various facet sizes

FEM simulations using COMSOL for a large range of facet sizes and emitter positions reveal a consistent drop-off in integrated emission intensity for emitter positions greater than 10 nm away from the centre of the NPoM cavity. Furthermore, the emission profile of a centred emitter is always a ring, and always evolves into a spot at a distance within 3 to 9 nm from centre. For facet sizes greater than 20 nm, there exists a limiting radius to the emitter position beyond which the emission profile reverts from spot to ring (around 12 nm for all facet sizes >20 nm). In all cases this reversion occurs for an emitter position with an integrated intensity <30% of that at the maximally out-coupled position.

The emitter position recovery procedure outlined in Fig.S2 depends on the facet size being known a priori, which can be extracted by suitably fitting the peak positions of the dark field scattering (eg Fig.4e with Fig.2a). From the $3 < r < 9$ nm region of Fig.S3, we see that, around w = 20 nm, changes in facet size of 10 nm result in far-field pattern shifts equivalent to dipole position shifts of at most 2 nm. We can therefore conclude that achieving an accuracy in the recovered emitter position of ± 1 nm requires knowledge of the facet size to within ± 5 nm, which gives easily measurable peak shifts in the dark field.

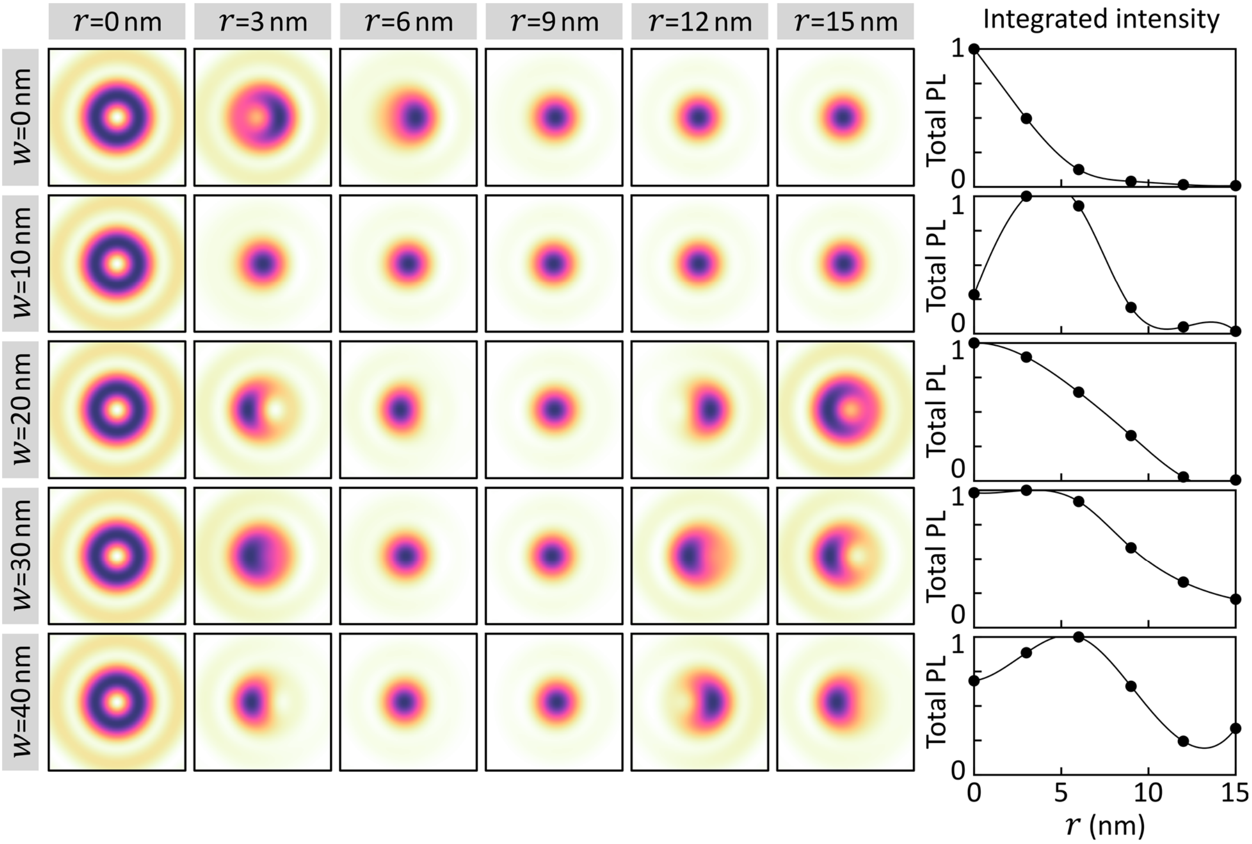

Fig.S3 | Simulations of emission profile at λ=660 nm for various facet sizes (left) and emitter positions (top). Integrated emission intensity (right) shown as a function of emitter position at each facet size.

Additional examples of NPoM emission profiles

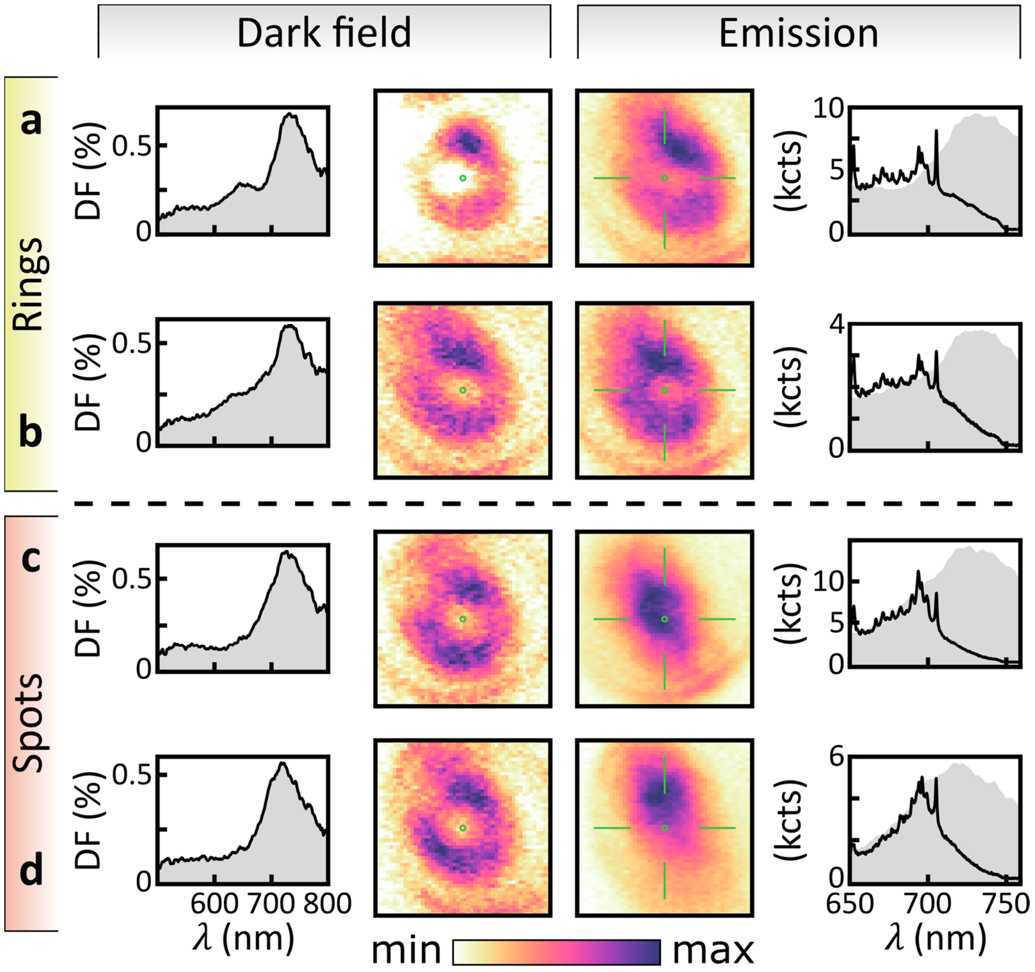

Fig.S4 | Additional examples of far-field emission profiles to compare with Fig.5. a, NPoMs with ring shaped emission and b, NPoMs with spot shaped emission.

DF Spectra

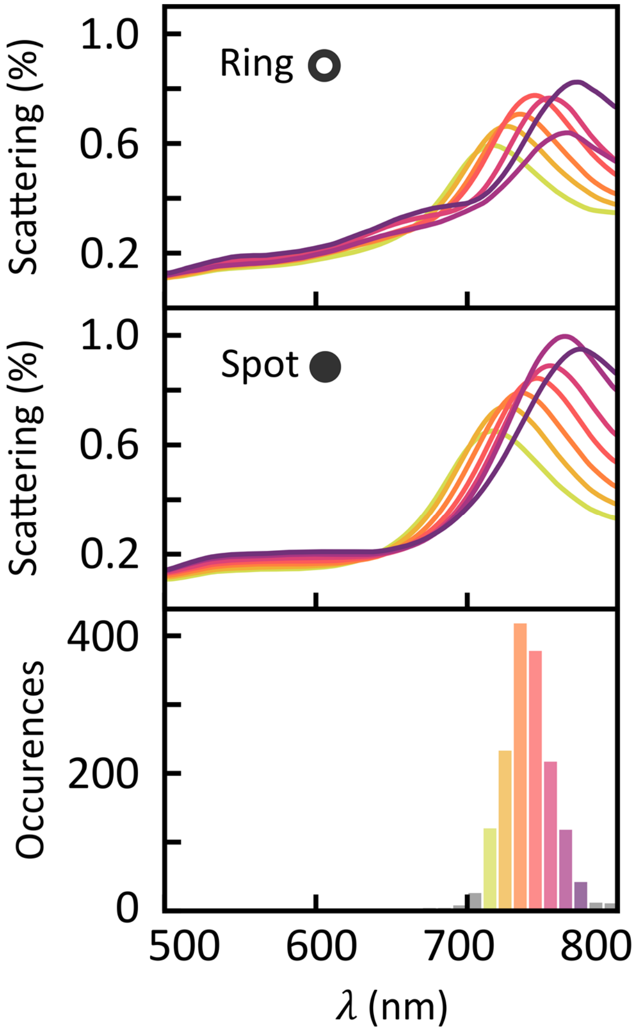

Fig.S5 | Averaged DF spectra of 1602 NPs, separated by light emission image shape into the 201 rings (top) and 1189 spots (middle), binned by the spectral position of their largest DF spectrum peak (bottom). Coloured lines are the averaged DF spectrum of NPs in the bin of the corresponding colour.

Emission from NPoMs prepared with CB[7] only

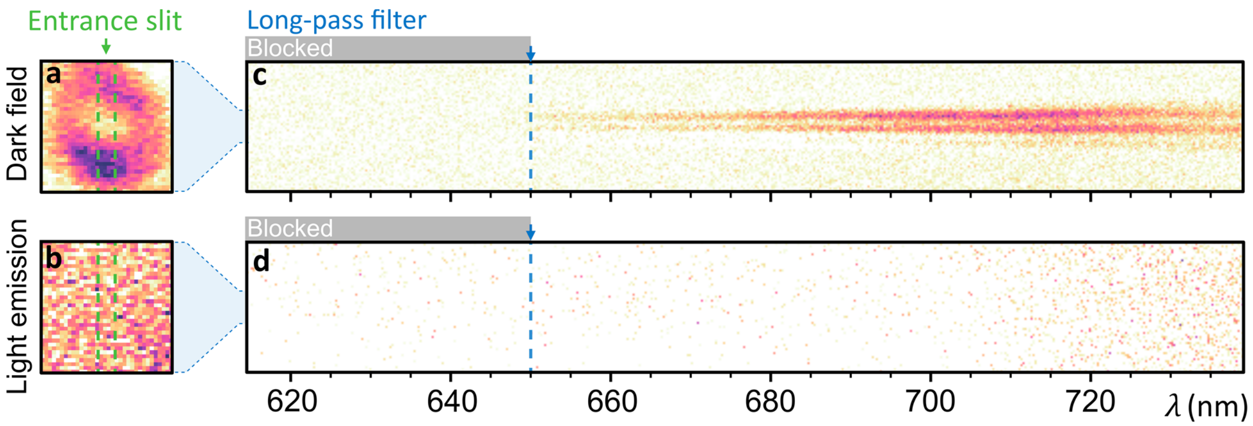

Fig.S6 | DF and light emission from a 80 nm Au NPoM with a molecular spacer consisting of CB[7] molecules without the addition of any dye molecules.

If a substrate of template stripped gold prepared initially with CB[7] but omitting the MB is used to build NPoMs from 80 nm gold nanoparticles using the usual drop-casting method, no emission is observed in most nanoparticles (supplementary Fig.S7a,c). The few particles in the sample that exhibit some form of weak emission have spot-like spatial distributions (supplementary Fig.S7b). This weak emission is most likely from electronic Raman scattering from the Au nanoparticle and it is 10-20 times weaker than the peak PL emission with dyes in the gap.

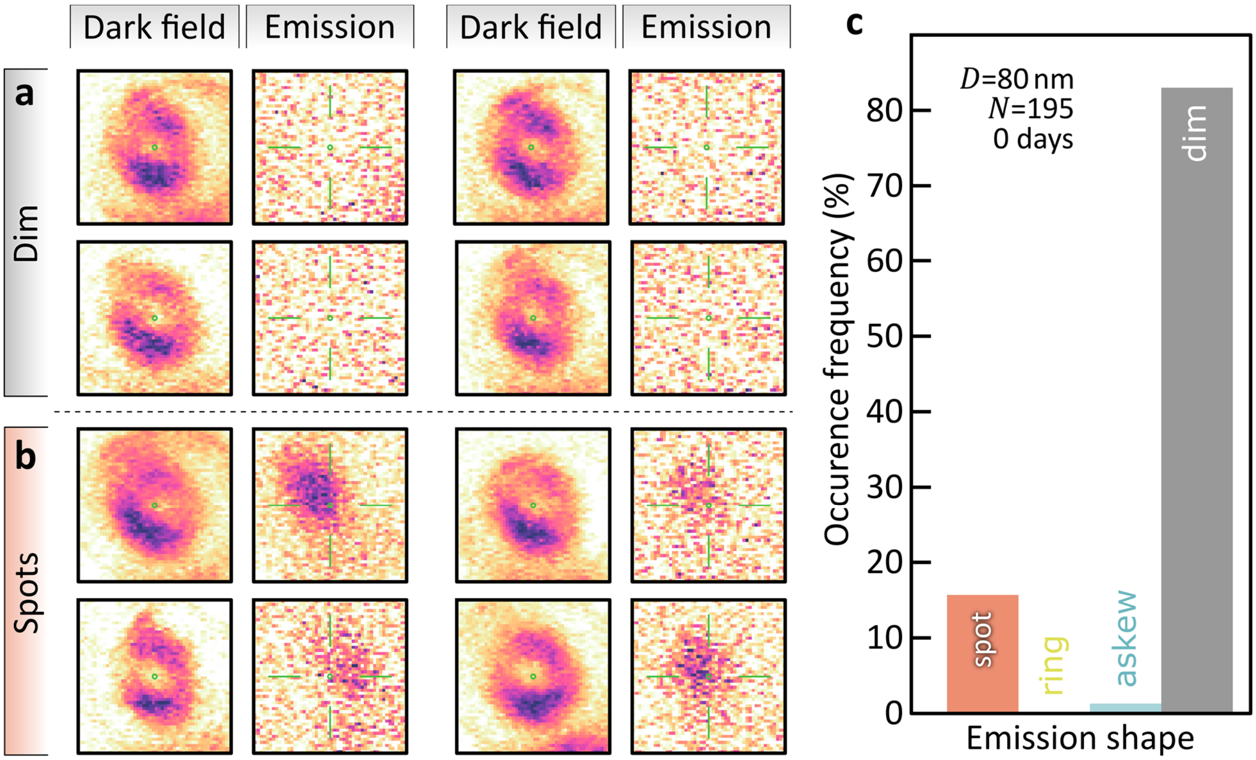

Fig.S7 | Far-field emission profiles of 80 nm Au NPoMs prepared with CB[7], but without MB dyes. a, Dark field and emission spatial profiles for four different nanoparticles exhibiting no emission or b, very weak spot-like emission. c, Percentage ratio of each distribution observed.

Time evolution of NPoM emission profile

An example of the time evolution of the intensity and angular distribution of NPoM emission was given in Fig.4. This evolution is subtly different for different NPoMs, with some exhibiting a greater or lesser number of revivals in emission intensity over time. This can be seen in the following additional examples (supplementary Fig.S8-10):

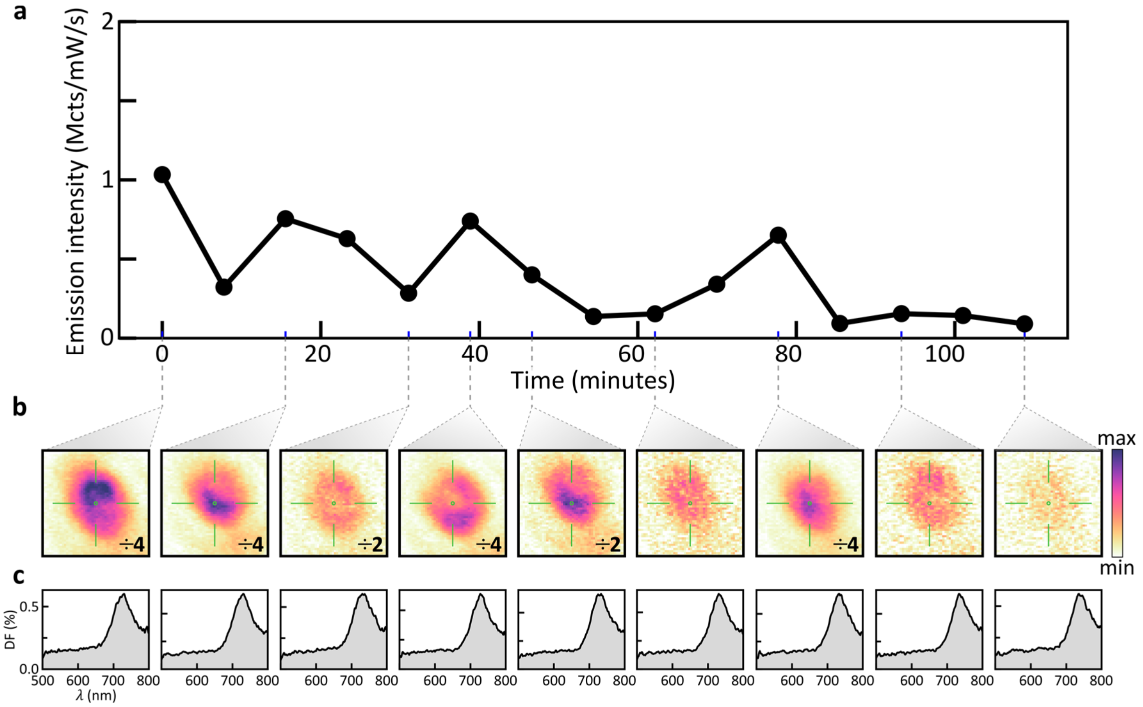

Fig.S8 | Time evolution of real-space emission from a single 80 nm NPoM (additional example #1). a, Integrated intensities vs time. b, Corresponding real-space spectrally-filtered emission images at times as marked, with the reticle showing the position of the dark-field ring centre. Integration times are 10 s, for 633 nm laser. c, DF spectra at the times marked in b.

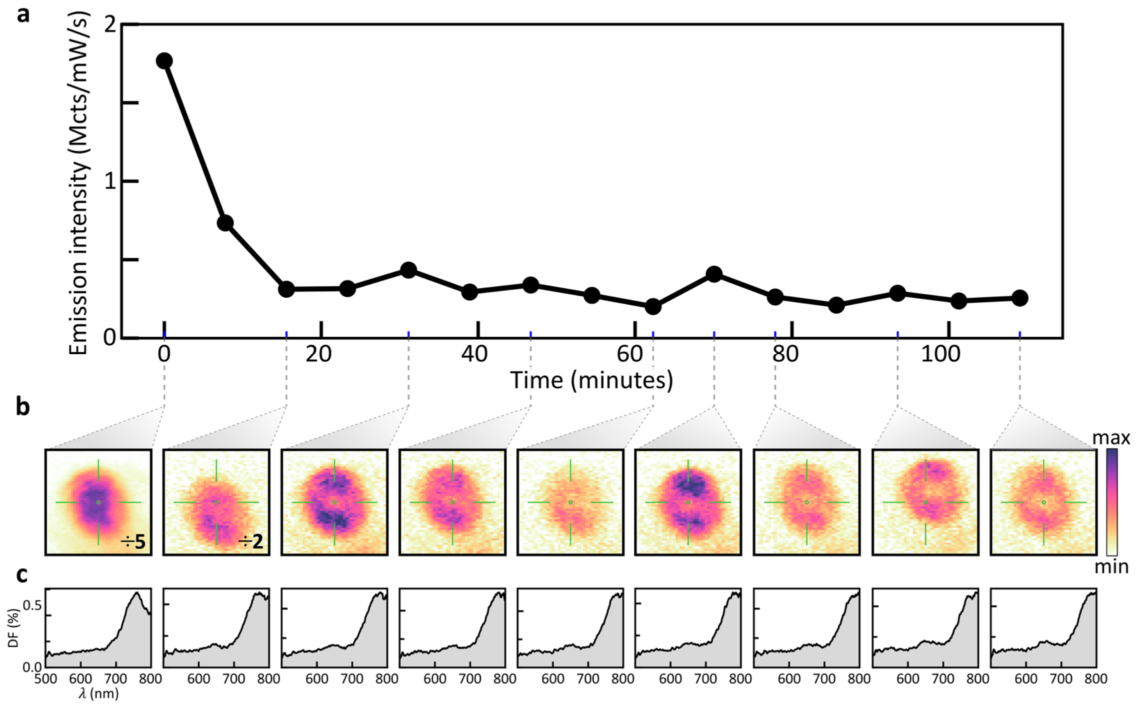

Fig.S9 | Time evolution of real-space emission from a single 80 nm NPoM (additional example #2). a, Integrated intensities vs time. b, Corresponding real-space spectrally-filtered emission images at times as marked, with the reticle showing the position of the dark-field ring centre. Integration times are 10 s, for 633 nm laser. c, DF spectra at the times marked in b.

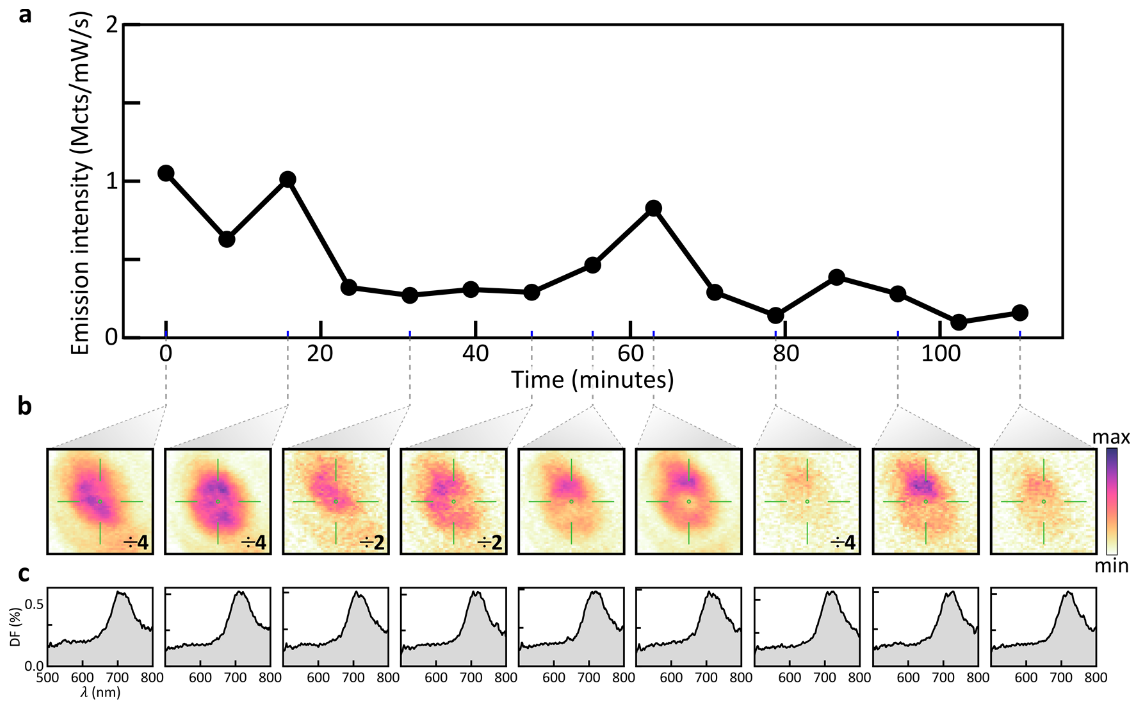

Fig.S10 | Time evolution of real-space emission from a single 80 nm NPoM (additional example #3). a, Integrated intensities vs time. b, Corresponding real-space spectrally-filtered emission images at times as marked, with the reticle showing the position of the dark-field ring centre. Integration times are 10 s, for 633 nm laser. c, DF spectra at the times marked in b.

Time evolution of emission spectra

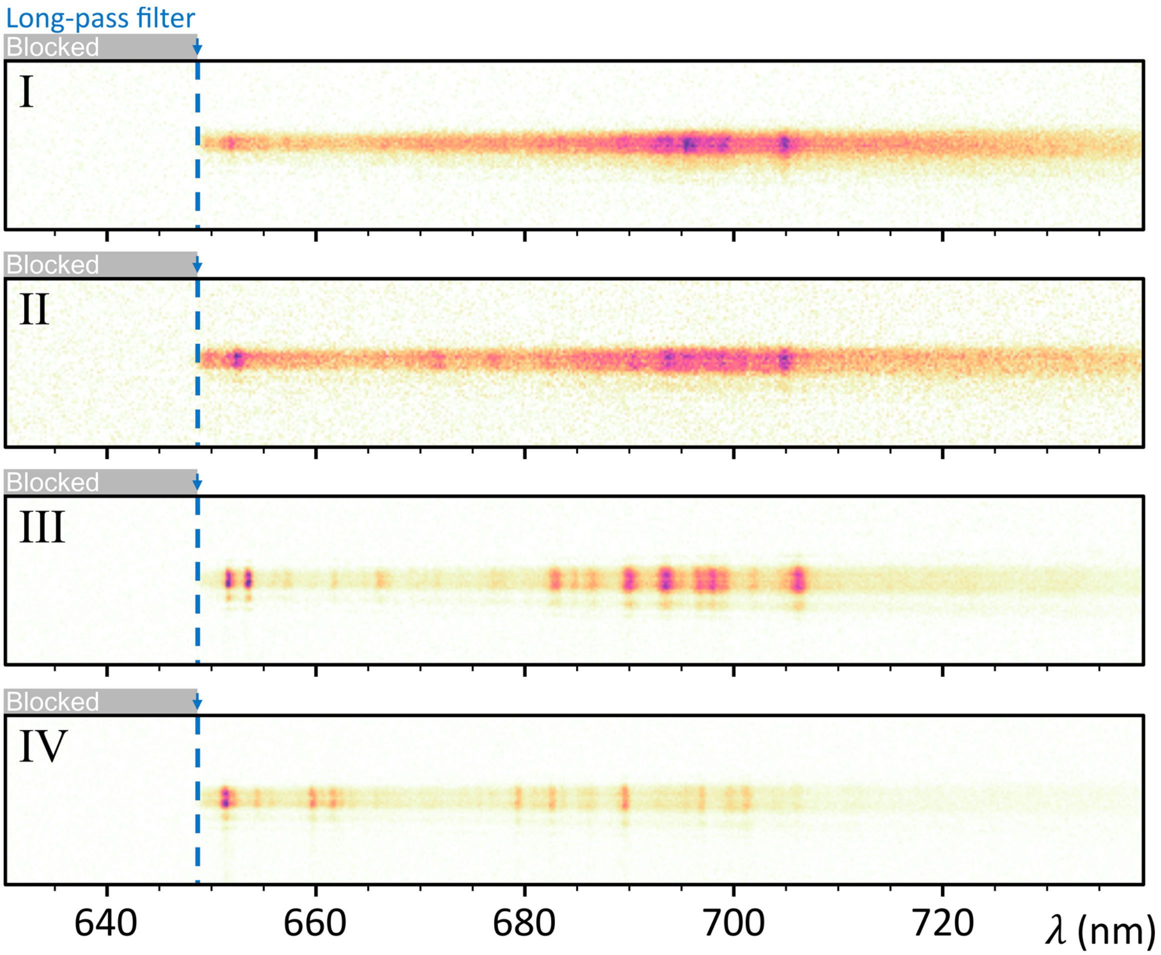

Fig.S11 | Evolution of emission spectra from a single NPoM under continuous illumination. Spectra shown are taken concurrently with the data in Fig.4, with I-IV corresponding to images & time given there. A 650 nm long-pass filter blocks all emission below 650 nm, including the excitation wavelength at 633 nm.

Supplementary Fig.S11 shows the emission spectra of the NPoM imaged in Fig.4 at four arbitrarily selected moments in time. Many of the spectral peaks observed over the course of observing this particle are consistent with the Raman spectrum of the MB dye, but shift in both position and intensity over time. One hypothesis to explain this is the migration of Au adatoms in the facet forming picocavities close to single molecules (1, 2) leading to a transient increase in the confinement of light to the molecule's vicinity. Another is the rearrangement of Au atoms within the NPoM facets (3) resulting in transient defects at the grain boundaries, which enhance the SERRS of nearby molecules (see main text discussion).

Simulations of multiple dipoles in a NPoM

Generating the far-field scattering profile for a NPoM hosting two or more molecular emitters within its plasmonic hot spot can be achieved by suitably combining the optical fields in the far-field from different emitters at different locations.

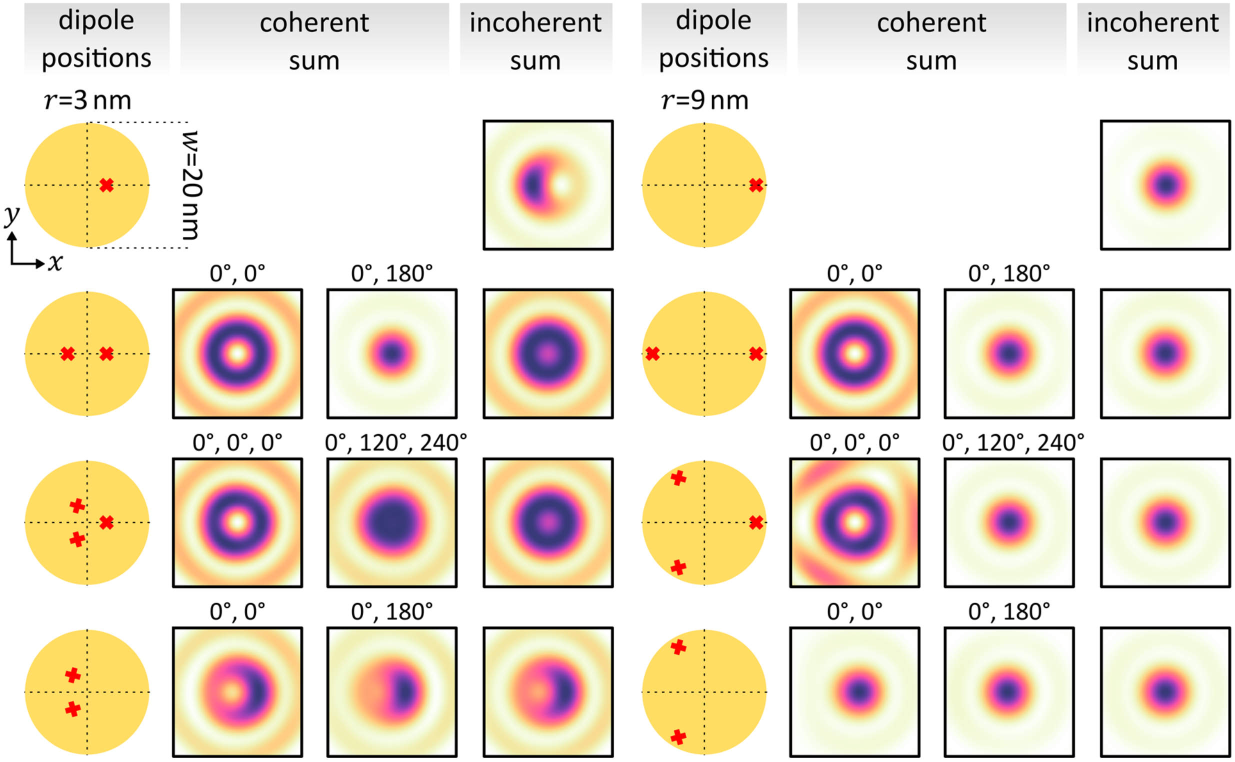

Fig.S12 | Resulting emission from 1,2,3 dipole places at r=3 or r=9 nm, when coherently summed with phases as marked, or incoherently summed.

Results of a few such combinations (Fig.S12) show how the asymmetric patterns are rapidly wiped out in both coherent and incoherent sums. As a result, it is not possible to uniquely quantify if the emitting multiple dipoles are coherently locked (as in a nanocavity polaritonic state) or independently incoherent.

Mode coupling at different radial positions

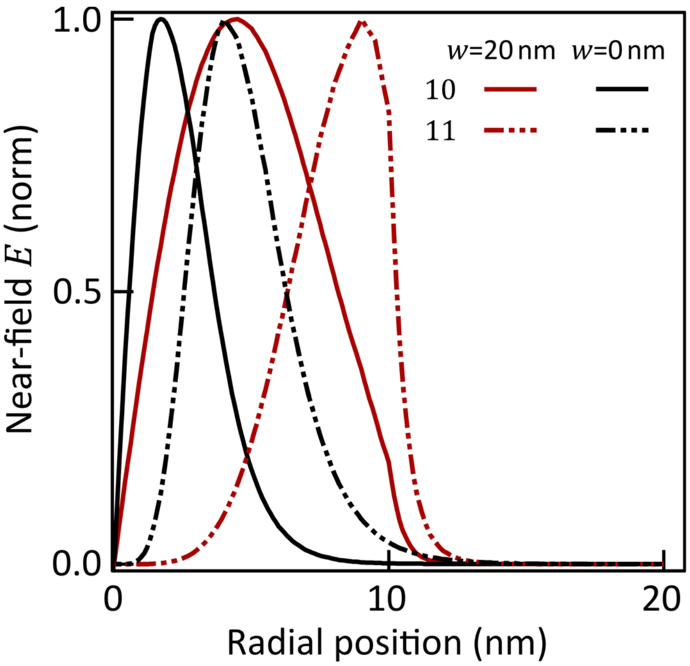

Fig.S13 | Normalised near-field mode amplitude density *vs* radial coordinate for the (10) and (11) modes in the gap of a 80 nm NPoM with either facet size of w=0 nm (black) or 20 nm (blue). This shows the integrated near-field amplitude at each radial position.

Preservation of samples under nitrogen

Experiments studying the number of each type of far-field scattering distribution are repeated using two different samples, to explore aging. The second sample gave the same result as the first, revealing no change in the numbers of each type (supplementary Fig.S14, compare with first sample in Fig.3g). The average integrated emission intensity of nanoparticles in this sample are also observed to be stable for the full duration of the experiments.

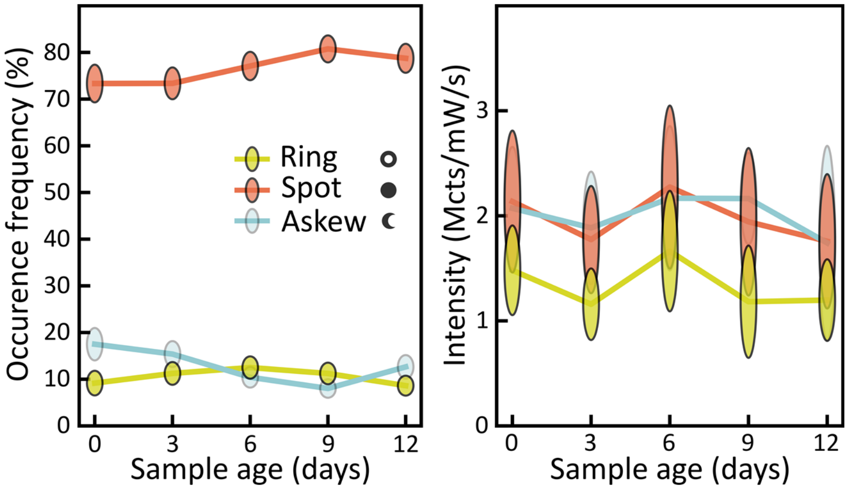

Fig.S14 | Long term time evolution of emission profile occurrence rates and integrated emission intensities for sample stored under nitrogen flow. The samples remain stable in storage for more than 12 days.

Emission and dark field spectra are likewise observed to be stable in samples stored for more than 12 days (supplementary Fig.S15).

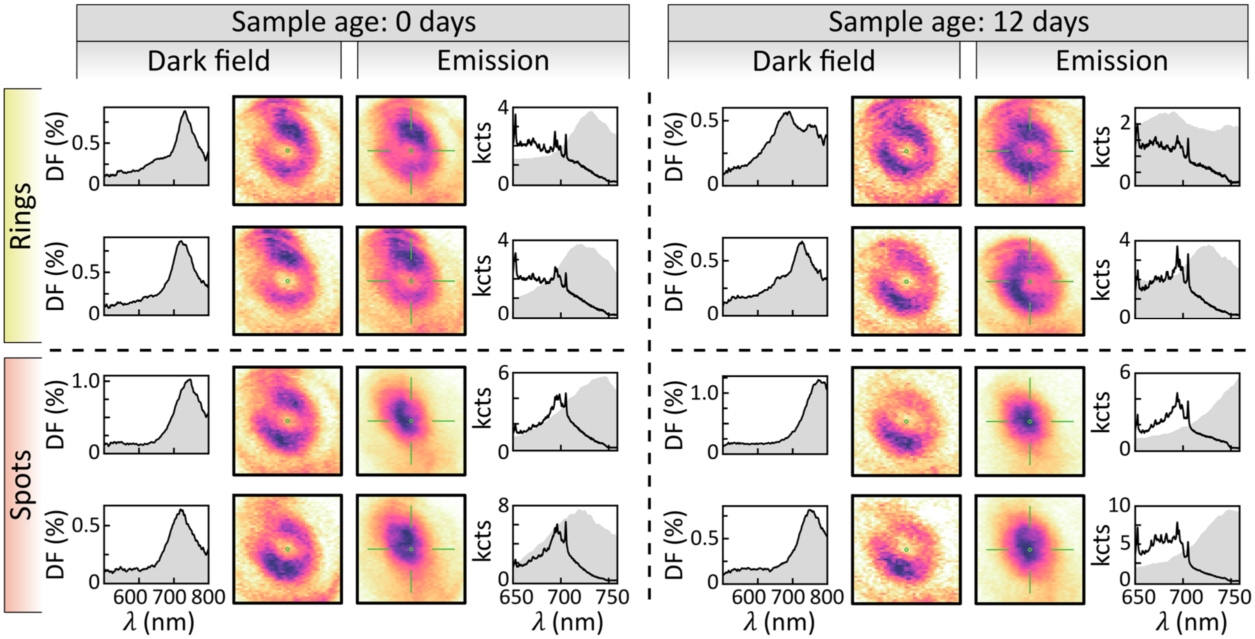

Fig.S15 | Dark field and emission spectra before and after 12 days storage.

Experimental setup

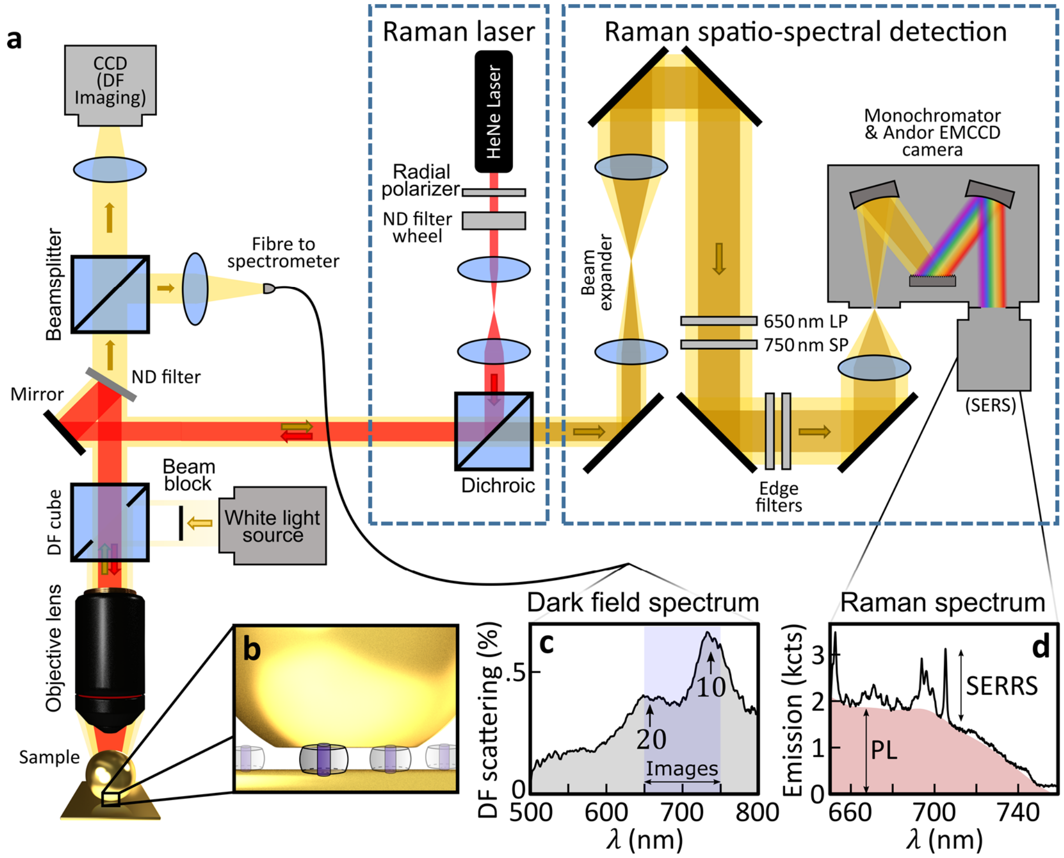

Fig.S16 | Experimental setup. a, Schematic of both imaging and spectroscopy. Left side shows imaging system used for alignment, automatic sample scanning, and collection of DF spectra. Boxed section 'Raman laser' shows radially-polarized 633 nm excitation laser used in collection of emission images and spectra. Boxed section 'Raman spatio-spectral detection' shows separation of emission from excitation, magnification stages, imaging, and collection of emission spectra. b, Schematic of typical NPoM, with a flat facet and a CB[7] spacer layer (not to scale). c, Dark field scattering spectrum of a typical NPoM with a ring-shaped emission and d, emission spectrum.

The experimental setup can be subdivided into three distinct parts. The first consists of an imaging microscope (Olympus BX51) equipped with a motorized stage (Prior Scientific H101) upon which the NPoM samples are placed. The samples are illuminated and imaged through a 100x bright field/dark field objective (Olympus, numerical aperture of 0.9), with either laser illumination or white light from a halogen lamp. A dark field (DF) cube and circular beam block are used in conjunction with the halogen lamp to illuminate the sample only at high angles for DF imaging. A reflective neutral density filter (Thorlabs ND503A, optical density of 0.3) is used in transmission as a broadband non-polarizing 50:50 beamsplitter. The ND filter directs part of the transmitted light to an imaging charge-coupled camera (Lumenera Infinity3-1) to view the sample, and to a fibre-coupled spectrometer (Ocean Optics QEPRO) for dark field spectroscopy. A cube beamsplitter (60:40 R:T) distributes light between these the imaging camera and the dark field spectrometer. The same reflective ND filter is used in reflection to both guide laser illumination towards the sample, and for directing emission towards the spatio-spectral detection system.

The second part of the setup is the Raman laser system used to excite the NPoM samples. It consists of an automatically shuttered HeNe laser (Thorlabs HNL210L, emission wavelength at 633 nm) directed through a radial polarization converter (Altechna RPC-632-04 S-waveplate). A computer controlled ND

filter wheel is used for power control, a beam expander with a magnification of 10x, and a dichroic beamsplitter used to direct the radially polarized laser towards the main experimental setup, and to direct collected emission towards the final part of the setup.

The spatio-spectral detection system consists of a telescope (magnification 10) formed with two plano-convex lenses. This is followed by a sequence of filters chosen to block the excitation wavelength and limit detection to the desired wavelength range by using a long pass filter (Thorlabs FEL0650, cut-off wavelength at 650 nm) and a short pass filter (Thorlabs FES0750, cut-off wavelength at 750 nm). After magnification and filtering, the remaining light is focused onto the slit of a monochromator (Andor Shamrock SR-303i), where it is directed to an electron-multiplying CCD camera (Andor Newton 970 EMCCD) using either the zero order of the grating monochromator if images are desired, or using the first diffraction order when spectra are desired.

Note: Effect of gap size on emission

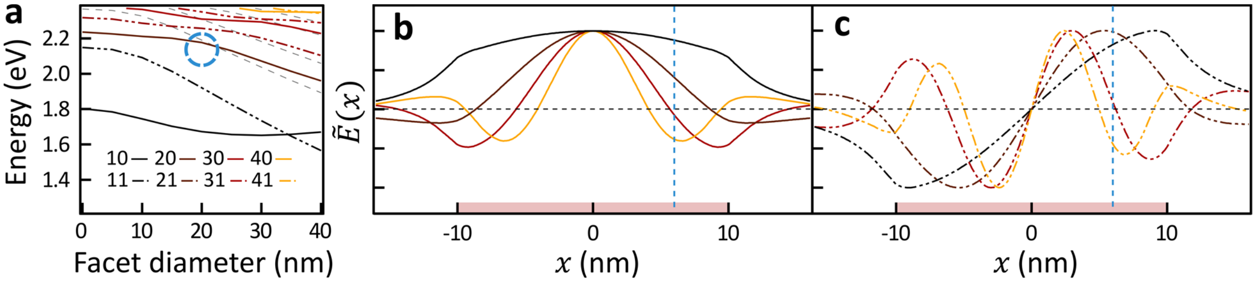

Fig.S17 | Plasmonic nanogap cavity modes of 80 nm NPoM with 2 nm gap and 20 nm facet diameter. a, Mode energies for the first 4 symmetric [solid lines (lm)=(10)-(40)] and asymmetric [dashed (11)-(41)] nanogap modes. Blue circle marks regime for facets of a typical D~80 nm NPoM. b,c, Corresponding near-field mode amplitudes with a 20 nm facet (normalised, colours as in a, dashed vertical at x=6 nm, extent of facet indicated by a pale red bar on the x-axis).

The CB[7] spacer layer used in the experimental portion of this paper precisely fixes the gap separation to 0.9±0.05 nm.(4) However, similar far field emission images are found theoretically for larger gap sizes. In such a case, the mode energy positions shift (see maps in (5), and Fig.S17), and thus so does the mode mixing. This is very similar to the use of different NP sizes (Fig.3e,f). Despite these changes, the evolution of the emission spatial image as the emitter is moved away from centre remains very similar (a ring is observed for a centred emitter, and emission becomes more spot-like as the emitter is shifted away from centre, as shown in Fig.S18). As a result, dye position reconstruction remains feasible for larger gap sizes.

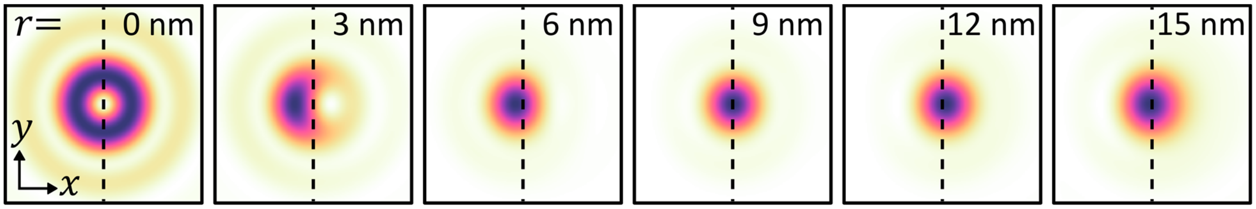

Fig.S18 | Far-field real-space images (normalized) simulated using FEM after collection through an 0.9NA objective, using the same structure and dimensions as in Fig.1b except with a 2 nm gap thickness instead of 1 nm.

Note: Mode nomenclature

In this article, the QNMs of the NPoM are labelled in order of increasing energy as lm = 10, 11, 20, 21, 22, 30, 31, 32, 33… where l and m are indices analogous to those of the spherical harmonics $Y_l^m(\theta, \phi)$ (see for example (6)). This nomenclature was chosen as it closely reflects the distribution of charge on the surface of a NPoM for each mode. Note the lm = 00 mode is intentionally left out as it represents the case of the NPoM accumulating or dissipating charge, which is not possible to excite for a NPoM on an electrically insulating spacer layer such as the CB:MB layers used here. Modes with even m radiate into a ring-shaped far-field spatial distribution, whereas modes with odd m radiate into spot-shaped far-field spatial distributions.

References

1. F. Benz, *et al.*, Single-molecule optomechanics in “picocavities.” *Science* **354**, 726–729 (2016).
2. M. Urbieta, *et al.*, Atomic-Scale Lightning Rod Effect in Plasmonic Picocavities: A Classical View to a Quantum Effect. *ACS Nano* **12**, 585–595 (2018).
3. Carnegie, C. et al., Flickering nm-scale disorder in a crystal lattice tracked by plasmonic ‘flare’ light emission. *in press Nat. Commun.* (2019).
4. B. de Nijs, *et al.*, Unfolding the contents of sub-nm plasmonic gaps using normalising plasmon resonance spectroscopy. *Faraday Discuss.* **178**, 185–193 (2015).
5. R. Chikkaraddy, *et al.*, How Ultranarrow Gap Symmetries Control Plasmonic Nanocavity Modes: From Cubes to Spheres in the Nanoparticle-on-Mirror. *ACS Photonics* **4**, 469–475 (2017).
6. P. Atkins, J. dePaula, *Physical Chemistry: Thermodynamics, structure, and change* (Macmillan Higher Education, 2014).
